# Succumbing to the Call of Violence – Sex-Linked Development of Appetitive Aggression in Relation to Familial and Organized Violence

**DOI:** 10.3389/fpsyg.2017.00751

**Published:** 2017-05-09

**Authors:** Mareike Augsburger, Danie Meyer-Parlapanis, Thomas Elbert, Corina Nandi, Manassé Bambonye, Anselm Crombach

**Affiliations:** ^1^Department of Psychology, University of KonstanzKonstanz, Germany; ^2^Department of Psychology, University of ZurichZurich, Switzerland; ^3^Department of Psychology, Université Lumière de BujumburaBujumbura, Burundi

**Keywords:** appetitive aggression, civil war, armed groups, combat, sex differences, gender, child abuse, child maltreatment

## Abstract

Appetitive aggression is the attraction to violent behavior, which can peak in the experience of a combat high. In various war and conflict scenarios, members of armed groups have reported developing a desire to hunt and even kill humans. More recently, we reported that the phenomenon has also been observed in female ex-combatants with varying participation in warfare. Despite recent investigations on risk factors for appetitive aggression, sex-specific pathways in the development of appetitive aggression have not yet been delineated. This study investigated moderation effects of sex on previously identified risk factors for appetitive aggression by means of regression analyses in a sample of individuals with varying degrees of warfare participation (overall sample, *n* = 602). First examining a sample characterized by backgrounds heterogeneous in both sociodemographic data and war experiences, the analysis was then replicated in a subsample of fighters active during the civil war (combatant sample, *n* = 109). In both samples, regression analyses revealed significant moderation effects of sex. Childhood maltreatment and traumatic events had positive associations on the development of appetitive aggression for males but a negative (childhood maltreatment) or no (traumatic events) association for females. Perpetrated events were more strongly correlated with appetitive aggression for females than for males. This pattern was pronounced for the combatant sample. These results are in favor of sex-linked pathways. In both sexes, appetitive aggression may have evolved as a biologically prepared response to cruel environments but might develop along different trajectories. The current study highlights the need for addressing appetitive aggression in order to support peace-building processes and emphasizes sex specific starting-points.

## Introduction

As of 2014 and at the exception of the genocide in Rwanda, the worldwide number of fatalities due to armed conflict has peaked since the end of the Cold War (Melander, 2015; Pettersson and Wallensteen, 2015). These violent conflicts span years and decades and often spiral into multiple, overlapping cycles of violence (World Bank, 2011). In the presence of ongoing conflict, individuals living amidst those insecure conditions are likely to develop an inclination toward aggression stemming from the potentially appealing aspects of violent behavior (Elbert et al., 2010, 2017; Weierstall and Elbert, 2011). In contrast to reactive types of aggression, i.e., a response to a perceived threat, appetitive aggression is intrinsically rewarding and associated with positively evaluated emotions such as excitement. For further distinction from other types of aggression, see Elbert et al. (2017).

The phenomenon of appetitive aggression has been reported by multiple combatant populations throughout the world, even years after the official conclusion of the armed conflicts (e.g., Hecker et al., 2012; Hermenau et al., 2013; Weierstall et al., 2013) and, to a lesser extent, in members of armed groups carrying out supportive, non-military tasks (Augsburger et al., 2015). While initially the attraction to violence was considered a potentially predominantly male phenomenon (Nell, 2006; Elbert et al., 2010), recent studies have revealed the occurrence of similar levels of appetitive aggression in both sexes involved in post-conflict regions (Augsburger et al., 2015; Meyer-Parlapanis et al., 2016). Appetitive aggression is likely to contribute to an elevated risk of rejoining an armed force after demobilization or a gang after a prison sentence (Maedl et al., 2010; Sommer et al., 2017). Accordingly, the identification of risk factors for high traits in appetitive aggression is essential in order to understand waves of instability in conflict regions.

Concerning the etiology of aggressive behavior and violent crime, researchers have focused on the role of adverse childhood experiences. Over two decades ago, Widom (1989) demonstrated that a history of child abuse manifests itself in one’s own perpetration of violence on subsequent generations, known as the cycle of violence. Several studies support this view (for a review see Maas et al., 2008). Additionally, exposure to lifetime traumatic events and subsequent symptoms of posttraumatic stress disorder increased the appeal of aggressive acts in military veterans (MacManus et al., 2013) and civilians (Reijneveld et al., 2003; Taft et al., 2010; Rasche et al., 2016). With respect to research in former conflict regions in Eastern Africa, maltreatment experienced by parents during their childhoods was a significant predictor of violence they later perpetrated against their own children (Crombach and Bambonye, 2015). War-affected individuals are especially vulnerable to passing experiences of maltreatment on to their own offspring (Catani, 2010).

Some researchers assumed sex-specific pathways in the transition from self-experienced childhood abuse toward aggressive behavior and crime. For instance, Cullerton-Sen et al. (2008) reported a greater risk of physical aggression after experiences of maltreatment for boys than for girls. Accordingly, in a longitudinal study both physical and emotional child maltreatment predicted adult crime but through different pathways for males and females (Lee et al., 2015). In addition, externalizing behaviors during childhood in response to child abuse were related to later crime commission for males only, whereas internalizing behaviors predicted female crime (Jung et al., 2015). In contrast, other studies found a direct association between exposure to family violence and subsequent proactive aggression only for adolescent females (Calvete and Orue, 2013). Accordingly, child sexual abuse was related to increased frequency of aggressive acts in women but not in men (Trabold et al., 2015). Moreover, others reported no sex differences for the relation between child abuse and adolescent delinquency or adult crime (Arata et al., 2007; Topitzes et al., 2012).

Regarding appetitive aggression specifically, there is evidence that combatants are drawn into a cycle of violence due to the intrinsically rewarding perpetration of aggression (Hecker et al., 2012; Weierstall et al., 2012). With respect to the impact of childhood maltreatment and traumatic events it was tested in a study with male Burundian former combatants and active soldiers. Childhood maltreatment strengthened the association between self-perpetrated violence and appetitive aggression. Lifetime traumatic events additionally predicted appetitive aggression (Nandi et al., 2015). Moreover, positive associations between appetitive aggression and former exposure to violence were evident in South African male adolescents (Hinsberger et al., 2016; Sommer et al., 2017).

Beyond, the identification of further risk factors for appetitive aggression has remained challenging and so far only been investigated in males. To date the hypothesis of sex-specific risk factors for the development of appetitive aggression has not been investigated. Accordingly, the purpose of the current study was to assess associations between risk factors in the development of appetitively aggressive behavior in males and females. We aimed to answer this question by testing moderation effects of sex on the prediction of appetitive aggression by previously identified risk factors, as moderation allows specifying conditions under which predictor and outcome are related (Hayes, 2013). More specifically, we investigated if sex moderated the association between appetitive aggression and perpetrated events, lifetime traumatic events, and childhood maltreatment respectively in a Burundian sample comprising of males and females with varying levels of adverse childhood experiences and war exposure and combat participation. For this reason, we chose to include civilians as well as combatants to reflect this wide array of diverse life experiences incurred during the civil war in Burundi. Accordingly, our analysis procedure was two-folded: In a first step, a mixed sample of Burundian male and female former members of armed groups and civilians were analyzed (overall sample). In a second step these results were validated in a sample comprising only of male and female former combatants having had similar experiences during fighting (combatant sample).

Burundi is a small country and located in the African Great Lakes region. Similar to its neighbor Rwanda, Burundi’s past is characterized by ethnic conflicts between Hutus and Tutsis, resulting in the death of over 300,000 civilians and a long-lasting civil war being waged until its formal termination in 2006 (Uvin, 2009). Despite the last official demobilizations of armed group members having taken place in 2009 (World Bank, 2009), the country continues to struggle with reinstating stability and security in the face of persistent outbreaks of violence (United States Department of State - Bureau of Democracy, Human Rights, and Labor, 2013). Burundi was targeted for data collection due to its violent history and present and persistent instability. Moreover, the known existence of a considerable number of female members of armed groups proved this country ideal for this investigation.

## Materials and Methods

### Procedure

This cross-sectional study combines data from two assessment periods in 2012 and 2014. Initially, research was undertaken with an almost exclusively male sample in 2012. At that time, the existence of female combatants was rather unknown, resulting in a limited number of female participants. In 2014 data collection was extended toward an entire group of female participants. Additionally, some of the most-affected male participants from the 2012 investigation could be interviewed a second time.

A team of experienced clinical psychologists from the University of Konstanz, Germany and advanced psychology students from the University Lumière in Burundi collected the data. Participation in the study was voluntary; all participants provided informed consent. They received financial compensation equivalent to 5aaa (2012, 2014) and a refund of transportation costs (2014). Ethical review boards of the University Lumière de Bujumbura, Burundi, and of the University of Konstanz, Germany, approved the project. Sample sizes were determined according to the feasibility of data collection in post-conflict regions. See the Supplementary Material for more detailed information about procedures.

### Participants

All individuals invited agreed to participate. In total semi-structured interviews were conducted with 605 Burundian participants. Three female combatants were excluded prior to data analysis due to discrepancies in the information provided.

The overall sample in the first analysis consisted of 453 former combatants (387 males, 66 females) who had been demobilized after the end of the civil war and 149 participants without combat experience (25 males, 124 females). The latter were either civilians or former members of armed groups having performed non-military tasks. In the second analysis all combatants assessed in 2014 were included, namely 53 female combatants and 56 male combatants who had been reassessed in 2014. Supplementary Figure 1 provides an overview of details regarding the assessment periods and the composition of the samples.

### Measures

Questionnaires used for semi-structured interviews were translated from validated English or French versions to Kirundi and blindly back translated. Potential divergences in meaning were discussed with all parties until consensus was reached. In addition to socio-demographic data and combat experience (yes/no), the following measures were applied:

#### Traumatic Events

To measure lifetime traumatic load, self-experienced (e.g., having survived a natural disaster) and witnessed (e.g., witnessed killing of someone) event types were assessed by means of a dichotomously coded event list with 19 items (response options: yes/no). Events included specific, war-related incidents and events of the Posttraumatic Diagnostic Scale (Foa et al., 1997). The event list has been applied in various war-affected populations in Africa (e.g., Neuner et al., 2004). Items were summed up to indicate the number of different experienced event types, ranging between 0 and 19. This checklist was applied in 2012 and 2014.

#### Perpetrated Events

Lifetime self-committed violence was assessed in the same manner as the traumatic load with a checklist previously used in combatant samples (Weierstall and Elbert, 2011). We assessed the lifetime perpetration of 14 different types of violence (e.g., physical assault, sexual assault, homicide). This checklist was applied in 2012 and 2014.

#### Childhood Maltreatment I

In both 2012 and 2014 abuse experienced before the age of 18 was assessed via four items covering the major domains of possible abuse (Teicher et al., 2006): sexual abuse, neglect, physical abuse, and frequent verbal abuse. As before, items were coded dichotomously, confirming occurrence during childhood, and summed up, ranging between 0 and 4. This checklist was applied in 2012 and 2014.

#### Childhood Maltreatment II

In order to further analyze the role of child maltreatment, a detailed checklist of 30 items was implemented in 2014. The checklist was derived from the domestic and community violence checklist, which had already been used with various samples of children in East Africa (Hermenau et al., 2011; Crombach and Elbert, 2014). It covers adverse childhood experiences from different domains including verbal, physical, and sexual abuse and neglect, and additionally assesses different degrees of intensity (e.g., being slapped, being hit with an object, being punched). The items refer to the first 18 years of the participant’s life. Answers were coded dichotomously (yes/no) and summed up to reach a total score ranging between 0 and 30. This checklist was applied to the participants in 2014 only.

#### Appetitive Aggression

A positive and exhilarating perception of violence was assessed using the 15-items Appetitive Aggression Scale (AAS). The AAS has been validated on 1,632 participants from war-affected regions. It presents good psychometric properties regarding validity and reliability. Factor analysis revealed one single factor, suggesting that it assesses a distinct construct of human aggression (Weierstall and Elbert, 2011). The AAS has been successfully applied in various post-crisis settings. Ratings were made on a five-point Likert scale from total disagreement (0) to total agreement (4) and summed up, reaching a sum score between 0 and 60. Cronbach α coefficient was 0.92 for the overall sample and 0.89 for the combatant sample. This checklist was applied in 2012 and 2014.

### Data Analysis

Using R, robust multiple linear regression analyses were applied to predict appetitive aggression (Rousseeuw et al., 2015). This approach allows dealing with the presence of non-normal and heteroscedastic data. In order to facilitate interpretation, continuous predictors were mean centered before entering into the regression equation (Hayes, 2013). Categorical variables *(sex, combat experience)* were dummy-coded with males and no combat experience as reference groups.

In a first step, previously identified risk factors (*combat experience*, *child maltreatment, perpetrated event types, traumatic event types*) and respective interactions between these variables and *sex* were entered into the regression model. Additionally, *age* and its interaction with *sex* were included as covariates. Subsequently, non-significant predictors and interactions except *sex* were removed from the model. Robust adjusted *r* squared was used as fit index. Robust Wald test with pseudo degrees of freedom was applied for model comparison.

## Results

In the overall sample (*n* = 412 males, *n* = 190 females), males were older (mean years 35.6, *SD* = 8.7 versus 30.4, *SD* = 7.9, *p* < 0.001), better educated [mean years = 6.6 (*SD* = 3.2), versus mean = 5.6 (*SD* = 3.9), *p* < 0.001], had fewer children [mean = 2.11 (*SD* = 2.1) versus mean = 2.8 (*SD* = 2.1), *p* < 0.001], had experienced more traumatic event types [mean = 13.6 (*SD* = 2.5) versus mean = 12.3, (*SD* = 3.8), *p* < 0.001] and had perpetrated more violent acts than females [mean = 8.1 (*SD* = 3.6), versus mean = 3.7 (*SD* = 4.3), *p* < 0.001]. Males also reported higher levels of appetitive aggression [mean = 27.4 (*SD* = 22.5), versus mean = 13 (*SD* = 13.6), *p* < 0.001]. Experienced maltreatment during childhood did not differ between the sexes [mean = 9 (*SD* = 1.0) versus mean = 1.1 (*SD* = 1.2), *p* = 0.27].

In the combatant sample (*n* = 56 males, *n* = 53 females), the sexes only differed in age and traumatic event types. Males were older than females [mean = 39.8 (*SD* = 10.03), versus mean = 31.0 (*SD* = 7.4), *p* < 0.001] and had experienced fewer traumatic event types [mean = 14.2 (*SD* = 2.3) versus mean = 15.9 (*SD* = 2.8), *p* < 0.001]. They had comparable levels of education [mean years males = 7.0 (*SD* = 2.8), mean years females = 6.5 (*SD* = 3.7), *p* = 0.52], and numbers of children [mean males = 3.1 (*SD* = 2.3), mean females = 2.6 (*SD* = 2.0), *p* = 0.26]. Experienced childhood maltreatment assessed with the more detailed questionnaire was similar [mean males = 14.8 (*SD* = 5.9), mean females = 15.5 (*SD* = 5.8), *p* = 0.73]. Also perpetrated events [mean males = 6.3 (*SD* = 3.3), mean females = 7.0 (*SD* = 4.1), *p* = 0.34] and appetitive aggression [mean males = 21.1 (*SD* = 9.1), mean females = 21.25 (*SD* = 11.7), *p* = 0.77] did not differ between the sexes in the combatant sample. Correlations between the variables are shown in **Table 1**.

**Table 1:** Correlation matrix grouped by sex for the overall sample **(A)** and the combatant sample **(B).**

	Age	CM I^a^/II^b^	Traumatic events	Perpetrated events	AAS Sum Score
**A. Overall sample (male/female)**					
Combat	0.21^∗∗∗^/-0.04	0.07/0.23^∗∗^	0.18^∗∗∗^/0.44^∗∗∗^	0.37^∗∗∗^/0.63^∗∗∗^	0.32^∗∗∗^/0.62^∗∗∗^
Age	–	-0.12^∗^/-0.13	-0.12^∗^/0.11	-0.15^∗∗^/0.02	-0.18^∗∗∗^/0.04
Child maltreatment I (CM I)^a^	–	–	0.21^∗∗∗^/0.34^∗∗∗^	0.23^∗∗∗^/0.29^∗∗∗^	0.25^∗∗∗^/0.19^∗∗^
Traumatic events	–	–	–	0.51^∗∗∗^/0.65^∗∗∗^	0.43^∗∗∗^/0.57^∗∗∗^
Perpetrated events	–	–	–	–	0.62^∗∗∗^/0.76^∗∗∗^
**B. Combatant Sample (male/female)**					
Age	–	-0.69^∗∗∗^/-0.35^∗^	-0.45^∗∗∗^/-0.21	-0.67^∗∗∗^/-0.21	-0.61^∗∗∗^/-0.15
Child maltreatment II (CM II)^b^	–	–	0.42^∗∗^/0.65^∗∗∗^	0.58^∗∗∗^/0.34^∗^	0.63^∗∗∗^/0.13
Traumatic events	–	–	–	0.35^∗∗^/0.4^∗∗^	0.35^∗∗^/0.29^∗^
Perpetrated events	–	–	–	–	0.5^∗∗∗^/0.75^∗∗∗^

*Spearman’s Rho rank correlations were calculated for continuous variables, point-biserial correlation for relations with the dichotomous variable (combat) in the overall sample. ^a^Refers to the four-item questionnaire of child abuse used in the total sample during the assessments in both 2012 and 2014; ^b^refers to the more detailed assessment during 2014 only. Asterisks indicate level of significance, with ^∗^*p* < 0.05, ^∗∗^*p* < 0.01, ^∗∗∗^*p* < 0.001*.

### Regression Analyses

Regarding the overall sample, all predictors and interactions except *sex* were significant (*child maltreatment I* and *sex* x *perpetrated events* only marginally). The covariate *age* and its interaction with *sex* also reached significance. Adjusted R squared was 0.63 (*SE* = 9.34), the model was superior to the null model with *F*(576,11) = 1358.3, *p* < 0.001.

Results were similar for the combatant sample from the 2014 study that utilized the more detailed child maltreatment questionnaire. However, neither *age* nor *traumatic events* nor their interactions had any impact on the prediction of appetitive aggression and consequently were removed from the model. Coefficients for *child maltreatment II* as well as the interaction *perpetrated events x sex* reached significance in the combatant sample. All other variables showed the same pattern as in the overall sample. Adjusted *R* squared was 0.52 (*SE* = 0.54), with *F*(103,5) = 161.03, *p* < 0.001. The regression models for both samples are shown in **Table 2**.

**Table 2:** Final regression models predicting appetitive aggression from a sex-linked pathway perspective for the overall sample (pattern A, left side) and the combatant sample (pattern B, right side).

	(A) Overall sample (*n* = 602)				(B) Combatants (*n* = 109)			
	*B*	95% CI	*SE*	*t*	*B*	95% CI	*SE*	*t*
Age	**-0.26^∗∗∗^**	[**-0.41, -0.11**]	**0.08**	**-3.94**				
Sex	2.60	[-1.20, 6.41]	1.94	1.34	-0.70	[-3.64, 2.24]	1.48	-0.47
Combat	**9.90^∗∗∗^**	[**6.44, 13.36**]	**1.76**	**5.62**		/	/	/
Child maltreatment ^a,b^	**1.30°**	[**-0.02, 2.62**]	**0.67**	**1.94**	**0.71^∗∗∗^**	**[0.46, 0.97]**	**0.13**	**5.56**
Perpetrated events	**1.81^∗∗∗^**	[**1.403, 2.21**]	**0.20**	**8.84**	**0.68^∗^**	**[0.12, 1.24]**	**0.28**	**2.42**
Traumatic events	**1.24^∗∗∗^**	[**0.69, 1.79**]	**0.28**	**4.4**				
Sex ^∗^ combat	**-5.69^∗^**	[**-10.63, -0.75**]	**2.51**	**-2.26**		/	/	/
Sex ^∗^ child maltreatment^a,b^	**-1.72^∗^**	[**-3.36, -0.07**]	**0.84**	**-2.05**	**-1.06^∗∗∗^**	**[-1.49, -0.62]**	**0.22**	**-4.82**
Sex ^∗^ perpetrated events	**0.54°**	[**-.07, 1.15**]	**0.31**	**1.75**	**1.66^∗∗∗^**	**[0.92, 2.40]**	**0.37**	**4.46**
Sex ^∗^ traumatic events	**-1.20^∗∗∗^**	[**-1.84, -0.56**]	**0.33**	**-3.67**				
Sex ^∗^ age	**0.28^∗∗^**	[**0.09, 0.47**]	**0.10**	**2.90**				

*The intercept was removed due to better readability. Unstandardized b-coefficients of the centered model with 95% confidence intervals, standard errors (*SE*) and *t*-values are shown. ^a^Refers to the four-item questionnaire of child abuse used in the total sample (child maltreatment I); ^b^refers to the more detailed assessment during 2014 (child maltreatment II). Asterisks indicate level of significance, with °*p* < 0.08, ^∗^*p* < 0.05, ^∗∗^*p* < 0.01, ^∗∗∗^*p* < 0.001. Significant coefficients are shown in boldface*.

**FIGURE 1:**
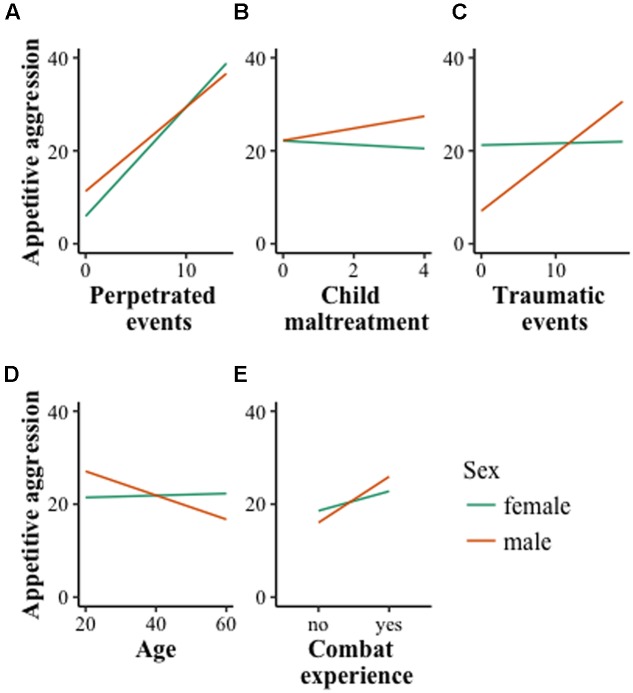
**Two-way interactions in the overall sample between sex and perpetrated events **(A)**, child maltreatment **(B)**, traumatic events **(C)**, age **(D)**, combat experience **(E)**, respectively.** Only four items were available for the overall sample to indicate childhood maltreatment. The figure demonstrates that, for females (green line), only self-perpetrated events and combat experience were associated with an increase in appetitive aggression, whereas for males, child maltreatment as well as traumatic events had an impact.

From **Figure 1** it can be derived that in the overall sample for females the association between *perpetrated events* and appetitive aggression was stronger than for males. There was a reverse effect for *combat experience*, with a stronger association existing for males. Moreover, *traumatic events* were correlated with appetitive aggression for males but not females. The same applied to the *age* of the participants, which was negatively related among males. Lastly, *child maltreatment* was only positively associated with appetitive aggression for males.

**FIGURE 2:**
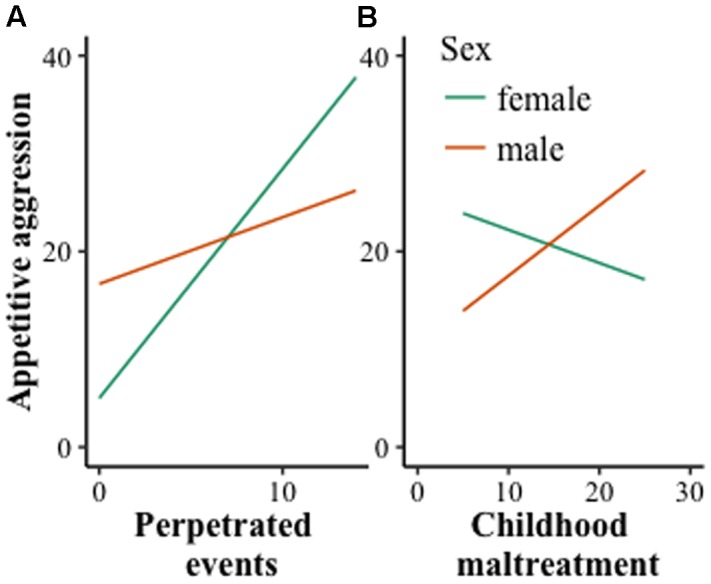
**Two-way interaction in the combatant sample between sex and perpetrated events **(A)**, and sex and childhood maltreatment **(B)**.** In this sample, childhood maltreatment was assessed with a detailed 30 item checklist. The figure shows that the pattern in the overall sample (see **Figure 1**) can be replicated in the combatant sample and with a different measurement regarding childhood maltreatment: For females, perpetrated events are even more strongly associated with appetitive aggression, whereas the reversed pattern illustrates that the association with childhood maltreatment is negative. Exposure to traumatic events was not relevant in this sample and removed from the figure.

Interaction effects of the combatant sample are visually displayed in **Figure 2**. They indicate that the interaction effect between perpetrated events and sex was even more pronounced in combatants. Additionally, traumatic events in the combatant sample were negatively associated with female appetitive aggression.

## Discussion

The aim of the current study was to investigate whether sex moderated relations between risk factors and appetitive aggression. To date, there has not yet been a study that has assessed appetitive aggression in a sample of war-affected individuals, including both civilians and combatants on active duty during the civil war.

Our results are in line with the assumption of sex-specific pathways: Both adverse childhood experiences and lifetime traumatic events had a positive association with appetitive aggression – but only for males. For females, there was no significant relation between appetitive aggression and traumatic events and a reversed relation between childhood maltreatment and appetitive aggression. This is concordant with previous studies showing positive associations between physical aggression and childhood abuse for males only (Cullerton-Sen et al., 2008). Moreover, our findings are supported by two longitudinal studies that demonstrate different trajectories for males and females in the transmission from child abuse to adult crime (Jung et al., 2015; Lee et al., 2015). However, our results also stand in contrast to a number of studies presenting a stronger positive association between adverse childhood experiences and aggression for females than for males (Calvete and Orue, 2013; Trabold et al., 2015) and with studies who did not find evidence for sex-specific pathways (Arata et al., 2007; Topitzes et al., 2012). These inconsistent findings with respect to sex-specific pathways in the development of aggression are likely to arise from several methodological and conceptual differences between the studies: In most retrospective studies, participants had been selected because of their involvement in the criminal justice system based on official documents, such as court records (Topitzes et al., 2012; Trabold et al., 2015). Accordingly, participants in those studies were limited to criminal offenders. In the single longitudinal study that did not find sex differences, participants were recruited from an institutional child welfare residence due to prior familial abuse experiences (Calvete and Orue, 2013). Accordingly, all of these studies were characterized by highly selective samples. In the current study, participants with a broad background of experiences were included, both civilians (who had also witnessed the Burundian civil war) and former members of armed groups, representing a less selective population. In addition, the majority of studies with a correlational design were with adolescent samples (Arata et al., 2007; Cullerton-Sen et al., 2008; Calvete and Orue, 2013). In the current study, participants were all adults. Consequently, pathways regarding adult aggression might be different.

Moreover, aggression-related outcome variables varied between the studies ranging from different types of aggressive behavior to crime or violent offending. Studies directly measuring criminal behavior such as specifically intimate partner violence (Trabold et al., 2015), acts of delinquency (Arata et al., 2007) or using official court records as outcome variable (Topitzes et al., 2012) were those that did not find evidence for sex-specific pathways. However, there is one exception: Lee et al. (2015) assessed different types of law-violating behavior over the year prior to the study and found sex-specific trajectories. Studies confirming findings about sex-specific pathways did not assess involvement in criminal activities but rather focused on externalizing behavior or aggressive acts (Cullerton-Sen et al., 2008; Jung et al., 2015), except for Calvete and Orue (2013), who did not support sex-specific pathways when assessing proactive aggression. Taken together, each of the studies differently conceptualized aggression or aggressive behavior. However, none specifically took into account appetitive aggression. Since this type of aggression seems to be an adaptation to violent environments (Crombach and Elbert, 2014) and develops under continuous threat (Hinsberger et al., 2016), its development is likely to differ from other types of aggression.

In summary, these conceptual variations between studies are likely to account for differences in findings and heterogonous results. Also, none of the previous studies were conducted in a post-crisis region such as Burundi with high rates of daily violence but rather in civilian Western European or North American settings. This fact might also contribute to differences in results. It is assumed that sex-specific trajectories might develop based on what type of aggressive behavior is measured in which setting. Regarding sex effects in appetitive aggression, it is likely to develop as an adaptation strategy under conditions of extreme stress and threat (Crombach and Elbert, 2014). From this point of view, sex-specific trajectories in long-term stress reactions and adaptation are advantageous. In explaining sex differences in general aggression, Campbell (2013) argues from an evolutionary perspective that males and females are thought to have participated differently in raising offspring. Accordingly, the survival of descendants may have relied to a greater extent on the mothers’ rather than the fathers’ survival. Thereby it is beneficial for mothers to be at a reduced risk for injury. Since lower levels of appetitive aggression reduce the overall inclination toward aggression and also subsequent risk of injury due to fighting (Augsburger et al., 2015), they increase the chances for reproductive success. Accordingly, from this evolutionary perspective, reducing females’ risk for developing appetitive aggression might be beneficial. As a consequence, behavioral changes in response to adverse childhood experiences or traumatic events may be associated with reduced risk for appetitively aggressive behavior in females but not in males. This interpretation also fits with previous research showing that females tend to employ less risky forms of aggressiveness more often, such as relational aggression (Archer, 2004; Cullerton-Sen et al., 2008). Furthermore, the risk of injury is reduced because relational aggression or indirect types of aggression do not typically involve high-risk physical forms of aggression. However when it is necessary to immediately protect one’s own offspring against a perceived threat, females are willing to risk high levels of injury – i.e., they demonstrate strong reactive aggressive reactions similar to those of males (Campbell, 2013). When the life threat persists, as is the case in post-conflict regions, we assume that there is a threshold shift in the perception of violence in which reactive aggression transitions to appetitive aggression in females. This interpretation aligns with our results, as lifetime perpetrated acts are more positively associated with appetitive aggression for females than for males. First, the perpetration of violent acts serve as the groundwork for the development of appetitive aggression to a greater extent in females. Thus, females can be carried away by the violence in a manner similar to males (Meyer-Parlapanis et al., 2016), but, in contrast to males, initial barriers for onset may be higher. Thus, it is not sex *per se* that compromises the ability to develop appetitive aggression but rather the interaction with specific experiences in order to enhance the probability of survival. Originating from these sex-specific behavioral reactions as suggested by Campbell (2013), we argue that in the context of ongoing threat high levels of appetitive aggression are also beneficial for females in order to maintain an approach-motivation to encounter the threat.

Regarding the interaction between age and sex in the overall sample, potential confounding variables such as the combat intensity associated with growing up during certain periods of the civil war are likely to be a reason for the moderation effects of sex. Furthermore, some female former members of armed groups performed non-military tasks during their service, complicating comparisons with the male, non-combatant group. To account for this potential confound, the overall model was proven in an exclusive sample of male and female former combatants assessed during the same time period (combatant sample). Despite the male former combatant group consisting of participants who had been interviewed again in 2014 due to high posttraumatic stress disorder related impairment and elevated levels of appetitive aggression, they did not significantly differ from female combatants in the aforementioned event types, indicating similar experiences shared overall between male and female combatants. This replication of the main results found in the overall sample in the combatant sample demonstrates robust sex linked effects in the development of appetitive aggression. Additionally, the more detailed assessment of childhood maltreatment with a different questionnaire in this second analysis adds even more weight to the stability of our findings.

### Limitations of the Study

Owing to the correlational nature of our research design, it is not possible to determine a cause-and-effect relationship. Moreover, effects might be underestimated due to the retrospective design with memory effects having affected the results. Also, our interpretation of the findings is suggestive in nature and needs further research in order to be verified. Additionally, types of traumatic events such as experiences of sexual violence are known to differ between the sexes and may thus be one of the drivers of the sex-specific trajectories, particularly in war-affected regions. This could not be taken into account in the analyses. Also, the assessment of child maltreatment with a more detailed questionnaire being utilized only in 2014 made it impossible to compare the effects of a different questionnaire in the overall sample. Lastly, numbers of female combatants (*n* = 66) in relation to male combatants (*n* = 387) in the overall analysis was very small, compromising generalizability of our findings.

## Conclusion

Our study substantially differs from other approaches regarding study design, specific sample, and setting. Whilst other studies took place in Western settings, we have focused on individuals in Burundi who have all faced war and crisis but each to very different extents (ranging from active participation within armed groups to witnessing the civil war as a civilian). Accordingly, the current sample was very heterogeneous, also with respect to potential sex-specific experiences during the civil war. Nevertheless, this initial investigation of sex-linked pathways in the development of aggression points to the possibility that appetitive aggression is fostered in conflict regions but along different trajectories for both sexes. Since appetitive aggression hinders the transition into a civilian society (Maedl et al., 2010), high traits should be addressed not only when targeting risk factors in demobilization and reintegration programs but also when providing general services for the civilian population within humanitarian settings in former crisis regions. Hereby, a sex-specific approach is strongly suggested. Moreover, future longitudinal studies should investigate sex-specific mechanisms in the development of appetitive aggression.

## Ethics Statement

This study was carried out in accordance with the recommendations of the ethical review boards of the University Lumière de Bujumbura, Burundi, and of the University of Konstanz, with written informed consent from all subjects. In case of illiteracy, oral informed consents were collected. All subjects gave written informed consent in accordance with the Declaration of Helsinki. The protocol was approved by the ethical review boards of the University Lumière de Bujumbura, Burundi, and of the University of Konstanz.

## Author Contributions

All authors conceived and designed the study. MA, DM-P, CN, and AC were responsible for data collection and project management. MA conducted statistical analyses and wrote the draft. All authors contributed to the interpretation of the data and critically reviewed the draft. All authors approved the final version of the manuscript for submission.

## Conflict of Interest Statement

The authors declare that the research was conducted in the absence of any commercial or financial relationships that could be construed as a potential conflict of interest.

## References

[B1] Arata C. M. Langhinrichsen-Rohling J. Bowers D. O’Brien N. (2007). Differential correlates of multi-type maltreatment among urban youth. *Child Abuse Negl.* 31 393–415. 10.1016/j.chiabu.2006.09.006 17412420

[B2] Archer J. (2004). Sex differences in aggression in real-world settings: a meta-analytic review. *Rev. Gen. Psychol.* 8 291–322. 10.1037/1089-2680.8.4.291.supp

[B3] Augsburger M. Meyer-Parlapanis D. Bambonyé M. Elbert T. Crombach A. (2015). Appetitive aggression and adverse childhood experiences shape violent behavior in females formerly associated with combat. *Front. Psychol.* 6:1756 10.3389/fpsyg.2015.01756 PMC4646969 26635666

[B4] Calvete E. Orue I. (2013). Cognitive mechanisms of the transmission of violence: exploring gender differences among adolescents exposed to family violence. *J. Fam. Viol.* 28 73–84. 10.1007/s10896-012-9472-y

[B5] Campbell A. (2013). “High stakes and low risks: Women and aggression,” in *A Mind of Her Own: The Evolutionary Psychology of Women*, ed. Campbell A. (Oxford: Oxford University Press). 10.1093/acprof:oso/9780199609543.001.0001

[B6] Catani C. (2010). War at home – a review of the relationship between war trauma and family violence. *Verhaltenstherapie* 20 19–27. 10.1159/000261994

[B7] Crombach A. Bambonye M. (2015). Intergenerational violence in Burundi: experienced childhood maltreatment increases the risk of abusive child rearing and intimate partner violence. *Eur. J. Psychotraumatol.* 6:26995 10.3402/ejpt.v6.26995 PMC4696461 26679146

[B8] Crombach A. Elbert T. (2014). The benefits of aggressive traits: a study with current and former street children in Burundi. *Child Abuse Negl.* 38 1041–1050. 10.1016/j.chiabu.2013.12.003 24411982

[B9] Cullerton-Sen C. Cassidy A. R. Murray-Close D. Cicchetti D. Crick N. R. Rogosch F. A. (2008). Childhood maltreatment and the development of relational and physical aggression: the importance of a gender-informed approach. *Child Dev.* 79 1736–1751. 10.1111/j.1467-8624.2008.01222.x 19037946 PMC3397662

[B10] Elbert T. Moran J. Schauer M. (2017). “Appetitive aggression,” in *Aggression and Violence: A Social Psychological Perspective*, ed. Bushman B. J. (New York, NY: Taylor & Francis/Routledge), 119–135.

[B11] Elbert T. Weierstall R. Schauer M. (2010). Fascination violence: on mind and brain of man hunters. *Eur. Arch. Psychiatry Clin. Neurosci.* 260(Suppl. 2), S100–S105. 10.1007/s00406-010-0144-8 20938671

[B12] Foa E. B. Cashman L. Jaycox L. Perry K. (1997). The validation of a self-report measure of posttraumatic stress disorder: the posttraumatic diagnostic scale. *Psychol. Assess.* 9 445–451. 10.1037/1040-3590.9.4.445

[B13] Hayes A. F. (2013). *Introduction to Mediation, Moderation, and Conditional Process Analysis: A Regression-Based Approach.* New York, NY: Guilford Press.

[B14] Hecker T. Hermenau K. Maedl A. Elbert T. Schauer M. (2012). Appetitive aggression in former combatants - Derived from the ingoing conflict in DR Congo. *Int. J. Law Psychiatry* 35 244–249. 10.1016/j.ijlp.2012.02.016 22420932

[B15] Hermenau K. Hecker T. Maedl A. Schauer M. Elbert T. (2013). Growing up in armed groups: trauma and aggression among child soldiers in DR Congo. *Eur. J. Psychotraumatol.* 4:21408 10.3402/ejpt.v4i0.21408 PMC3820919 24224078

[B16] Hermenau K. Hecker T. Ruf M. Schauer E. Elbert T. Schauer M. (2011). Childhood adversity, mental ill-health and aggressive behavior in an African orphanage: changes in response to trauma-focused therapy and the implementation of a new instructional system. *Child Adolesc. Psychiatry Ment. Health* 5:29 10.1186/1753-2000-5-29 PMC3189861 21943214

[B17] Hinsberger M. Sommer J. Kaminer D. Holtzhausen L. Weierstall R. Seedat S. (2016). Perpetuating the cycle of violence in South African low-income communities: attraction to violence in young men exposed to continuous threat. *Eur. J. Psychotraumatol.* 7:29099 10.3402/ejpt.v7.29099 PMC4706593 26747683

[B18] Jung H. Herrenkohl T. I. Lee J. O. Hemphill S. A. Heerde J. A. Skinner M. L. (2015). Gendered pathways from child abuse to adult crime through internalizing and externalizing behaviors in childhood and adolescence. *J. Interpers. Viol.* 10.1177/0886260515596146 [Epub ahead of print]. PMC4991959 26264725

[B19] Lee J. O. Herrenkohl T. I. Jung H. Skinner M. L. Klika J. B. (2015). Longitudinal examination of peer and partner influences on gender-specific pathways from child abuse to adult crime. *Child Abuse Negl.* 47 83–93. 10.1016/j.chiabu.2015.07.012 26271556 PMC4567933

[B20] Maas C. Herrenkohl T. I. Sousa C. (2008). Review of research on child maltreatment and violence in youth. *Trauma Viol. Abuse* 9 56–67. 10.1177/1524838007311105 18182631

[B21] MacManus D. Dean K. Jones M. Rona R. J. Greenberg N. Hull L. (2013). Violent offending by UK military personnel deployed to Iraq and Afghanistan: a data linkage cohort study. *Lancet* 381 907–917. 10.1016/s0140-6736(13)60354-2 23499041

[B22] Maedl A. Schauer E. Odenwald M. Elbert T. (2010). “Psychological rehabilitation of ex-combatants in Non-Western, post-conflict settings,” in *Trauma Rehabilitation after War and Conflict: Community and Individual Perspectives*, ed. Martz E. (New York, NY: Springer), 177–214. 10.1007/978-1-4419-5722-1_9

[B23] Melander E. (2015). *Organized Violence in the World 2015: An Assessment by the Uppsala Conflict Data Program.* Uppsala: Uppsala University.

[B24] Meyer-Parlapanis D. Weierstall R. Nandi C. Bambonye M. Elbert T. Crombach A. (2016). Appetitive aggression in women: comparing male and female war combatants. *Front. Psychol.* 6:1972 10.3389/fpsyg.2015.01972 PMC4700207 26779084

[B25] Nandi C. Crombach A. Bambonye M. Elbert T. Weierstall R. (2015). Predictors of posttraumatic stress and appetitive aggression in active soldiers and former combatants. *Eur. J. Psychotraumatol.* 6:26553 10.3402/ejpt.v6.26553 PMC4408319 25908529

[B26] Nell V. (2006). Cruelty’s rewards: the gratifications of perpetrators and spectators. *Behav. Brain Sci.* 29 211–257. 10.1017/S0140525X06009058 17214016

[B27] Neuner F. Schauer M. Karunakara U. Klaschik C. Robert C. Elbert T. (2004). Psychological trauma and evidence for enhanced vulnerability for posttraumatic stress disorder through previous trauma among West Nile refugees. *BMC Psychiatry* 4:34 10.1186/1471-244X-4-34 PMC529265 15504233

[B28] Pettersson T. Wallensteen P. (2015). Armed conflicts, 1946-2014. *J. Peace Res.* 52 536–550. 10.1177/0022343315595927

[B29] Rasche K. Dudeck M. Otte S. Klingner S. Vasic N. Streb J. (2016). Factors influencing the pathway from trauma to aggression: a current review of behavioral studies. *Neurol. Psychiatry Brain Res.* 22 75–80. 10.1016/j.npbr.2016.01.009

[B30] Reijneveld S. A. Crone M. R. Verhulst F. C. Verloove-Vanhorick S. P. (2003). The effect of a severe disaster on the mental health of adolescents: a controlled study. *Lancet* 362 691–696. 10.1016/s0140-6736(03)14231-6 12957091

[B31] Rousseeuw P. Croux C. Todorov V. Ruckstuhl A. Barrera-Salibian M. Verbeke T. (2015). *Robustbase: Basic Robust Statistics [Software].* Available at: http://cran.r-project.org/package=robustbase

[B32] Sommer J. Hinsberger M. Elbert T. Holtzhausen L. Kaminer D. Seedat S. (2017). The interplay between trauma, substance abuse and appetitive aggression and its relation to criminal activity among high-risk males in South Africa. *Addict. Behav.* 64 29–34. 10.1016/j.addbeh.2016.08.008 27540760 PMC5102240

[B33] Taft C. T. Schumm J. Orazem R. J. Meis L. Pinto L. A. (2010). Examining the link between posttraumatic stress disorder symptoms and dating aggression perpetration. *Violence Vict.* 25 456–469. 10.1891/0886-6708.25.4.456 20712145

[B34] Teicher M. H. Samson J. A. Polcari A. McGreenery C. E. (2006). Sticks, stones, and hurtful words: relative effects of various forms of childhood maltreatment. *Am. J. Psychiatry* 163 993–1000. 10.1176/ajp.2006.163.6.993 16741199

[B35] Topitzes J. Mersky J. P. Reynolds A. J. (2012). From child maltreatment to violent offending: an examination of mixed-gender and gender-specific models. *J. Interpers. Violence* 27 2322–2347. 10.1177/0886260511433510 22279130 PMC3797999

[B36] Trabold N. Swogger M. T. Walsh Z. Cerulli C. (2015). Childhood sexual abuse and the perpetration of violence: the moderating role of gender. *J. Aggress. Maltreat. Trauma* 24 381–399. 10.1080/10926771.2015.1022288

[B37] United States Department of State - Bureau of Democracy Human Rights and Labor (2013). *Burundi 2013 Human Rights Report.* Available at: http://www.state.gov/j/drl/rls/hrrpt/humanrightsreport/index.htm?year=2013\&dlid=220088

[B38] Uvin P. (2009). *Life After Violence. A Peoples Story of Burundi.* NewYork, NY: Zed books.

[B39] Weierstall R. Elbert T. (2011). The Appetitive Aggression Scale-development of an instrument for the assessment of human’s attraction to violence. *Eur. J. Psychotraumatol.* 2:8430 10.3402/ejpt.v2i0.8430 PMC3402137 22893817

[B40] Weierstall R. Haer R. Banholzer L. Elbert T. (2013). Becoming cruel: appetitive aggression released by detrimental socialisation in former Congolese soldiers. *Int. J. Behav. Dev.* 37 505–513. 10.1177/0165025413499126

[B41] Weierstall R. Schalinski I. Crombach A. Hecker T. Elbert T. (2012). When combat prevents PTSD symptoms–results from a survey with former child soldiers in Northern Uganda. *BMC Psychiatry* 12:41 10.1186/1471-244X-12-41 PMC3413590 22583755

[B42] Widom C. S. (1989). The cycle of violence. *Science* 244 160–166. 10.1126/science.2704995 2704995

[B43] World Bank (2009). *Burundi - Emergency Demobilization, Reinsertion and Reintegration Project.* Washington, DC: Author.

[B44] World Bank (2011). *World Development Report. Conflict, Security, and Development.* Washington DC: Author.

