# Supplementary material for: Succumbing to the Call of Violence – Sex-Linked Development of Appetitive Aggression in Relation to Familial and Organized Violence
Source: Front Psychol. 2017 May 9;8:751. doi: 10.3389/fpsyg.2017.00751 (PMC5422546; doi:10.3389/fpsyg.2017.00751)
Supplement: Supplementary file 1 [file Data_Sheet_1.docx]

Supplementary Material

Surrendering to the call of violence – Sex-linked biographical influences on the development of appetitive aggression

Augsburger, M.^*^, Meyer-Parlapanis, D., Elbert, T. , Nandi, C., Bambonye, M., Crombach, A.

*** Correspondence:** Mareike Augsburger: mareike.augsburger@uni-konstanz.de

# Supplementary Information regarding procedures

# Students had received a multiple weeks of training in psychological diagnostics and concepts of aggression and traumatic stress. Clinical psychologists conducted first interviews with local students as translators (Kirundi – French or English). Local students conducted subsequent interviews under continuous on-site supervision; a personal debriefing and discussion regarding ratings together with a clinical psychologist took place after the end of each interview. Places for data collection varied depending on the external circumstances such as security concerns, and took place at University Lumière de Bujumbura (in 2012), at the Centre for Mental Health of the Burundian army (in 2014), and at training facilities provided by the Burundian army (in 2012 and 2014). All interviewers ensured a private atmosphere, devoid of third party observation. Former members of armed groups were contacted through the local veteran’s association; civilians were recruited from the same living quarters in and around Bujumbura, the capital of Burundi. By means of this approach, participants were individuals with different levels of combat participation who were all living in similar socioeconomic conditions. Inclusion criteria for all participants were legal age, no signs of acute drug or alcohol intoxication and previous membership in one of Burundi’s armed groups for former members, respectively no previous affiliation for civilians.

# Supplementary Figure

**Assessment 2012**

n = 25 male non-combatants

n = 20 female non-combatants

n = 15 female combatants

n = 387 male combatants

**Assessment 2014**

n = 51/53 female combatants^a^

n = 104 female non-combatans

n = 56 male combatants^b^

**Supplementary Figure 1.** Composition of the two samples. Dark gray shaded areas refer to the overall sample, light grey shaded fields refer to the combatant sample. ^a)^ Two female participants from 2014 were excluded from the overall analysis because they had aready participated in 2012. ^b)^ 56 male combatants from 2012 with highest scores of both appetitive aggression and posttraumatic stress disorder were again interviewed in 2014.
